# “When one has no REAL illness”—analysis of the knowledge component of mental health literacy in children and adolescents of parents with a mental illness

**DOI:** 10.3389/fpsyt.2024.1423326

**Published:** 2024-07-30

**Authors:** Lina Kinzenbach, Katharina Praum, Markus Stracke, Christina Schwenck, Meinhard Kieser, Kathleen Otto, Corinna Reck, Ricarda Steinmayr, Linda Wirthwein, Anna-Lena Zietlow, Hanna Christiansen, the COMPARE-family Research Group

**Affiliations:** ^1^ Department of Psychology, Clinical Child and Adolescent Psychology, Philipps-University Marburg, Marburg, Germany; ^2^ Department of Clinical Child and Adolescent Psychology, Justus Liebig University Giessen, Giessen, Germany; ^3^ Institute of Medical Biometry, University of Heidelberg, Heidelberg, Germany; ^4^ Department of Work and Organizational Psychology, Philipps-University Marburg, Marburg, Germany; ^5^ Department of Psychology, Ludwig-Maximilians-Universität München, Munich, Germany; ^6^ Department of Psychology, Technical University Dortmund, Dortmund, Germany; ^7^ Department of Psychology, Clinical Child and Adolescent Psychology, Technische Universität Dresden, Dresden, Germany

**Keywords:** mental health literacy, children of parents with a mental illness, general knowledge about mental disorders, specific disorder knowledge, deductive qualitative approach

## Abstract

**Introduction and objective:**

Mental Health Literacy (MHL) is important in promoting youth mental health. One key aspect of MHL is knowledge about mental disorders, which is particularly relevant for populations at risk for developing mental disorders, such as children of parents with a mental illness (COPMI), representing a mechanism within the transgenerational transmission. Currently, COPMI’s level of disorder knowledge in general, and about the specific parental disorder has not been comprehensively researched. We, therefore, aimed to assess COPMI’s disorder knowledge and clarify its association with COPMI’s age and sex exploratively. To assess both general and disorder-specific knowledge, we took a novel approach that makes disorder knowledge comparable across samples and over time.

**Methods:**

A mixed method analysis of N = 181 semi-structured MHL interviews with COPMI (aged 5 to 17 years) was carried out in the COMPARE—family study in Germany. We conducted a DSM-oriented deductive qualitative content analysis to assess COPMI’s general and specific disorder knowledge. Chi-square tests served to identify age and sex differences.

**Results:**

Children revealed limited knowledge of mental disorders in general, whereas adolescents displayed more knowledge that was also partly consistent with descriptions of classification systems like the DSM-5. The level of specific knowledge about the parent’s disorder depended on the disorder group. More children displayed adequate knowledge of somatic and anxiety disorders compared to trauma and depressive disorders, and more adolescents displayed adequate knowledge of depressive and anxiety disorders. COPMI’s age and sex were found to be significantly associated with disorder knowledge: adolescents exhibited higher levels of adequate general and specific disorder knowledge, and males exhibited higher levels of adequate general disorder knowledge.

**Conclusion:**

Assessing COPMI’s disorder knowledge and identifying associated age and sex differences yield valuable insights into the knowledge component of the MHL theory. Our findings can help to improve psychoeducational interventions for COPMI by orienting them to their prevailing levels of disorder knowledge. We recommend employing and extending the DSM-oriented deductive approach to assess knowledge within MHL. Analyses involving additional assessments within the COMPARE—family study are in preparation to identify potential knowledge gains over time, and associations to COPMI’s own well-being and mental health symptoms.

## 1 Introduction

Nearly two-thirds of mental disorders have their onset before the age of 25 years (1) making them one of the leading causes of health impairment in children and adolescents (2). Mental disorders exert a profound impact on social, emotional, and cognitive development (3) highlighting the critical need for effective prevention strategies in youth populations. This is particularly important for populations already carrying a higher risk to develop a mental disorder, such as children of parents with a mental illness [COPMI (4)]. COPMI face a significantly heightened lifetime risk for developing any mental disorder, namely, 55%, compared to children of healthy parents, who have a 14% lifetime risk for developing any mental disorder (5).

In the transgenerational transmission of mental disorders, COPMI’s lack of knowledge about the parental mental disorder has been identified as one important risk factor (6, 7). Knowledge about mental disorders serves as a basis for understanding mental illness and is part of the broader concept of mental health literacy (MHL). MHL was first defined by Jorm et al. (8) as knowledge and beliefs about mental disorders for their recognition, management, or prevention. More recent definitions, for example, by Kutcher et al. (9), focus on four main aspects of MHL as follows: (1) understanding how to acquire and maintain good mental health, (2) understanding mental disorders and their treatments, (3) reducing the stigma related to mental disorders, and (4) increasing help-seeking efficacy.

Promoting MHL among children and adolescents holds significant promise to foster their resilience, reducing the burden of mental disorders and mitigating adverse outcomes associated with mental disorders (10). This is evident in findings indicating that adolescents with high levels of MHL are less likely to experience psychological distress (11), more likely to seek help in case of mental impairments, and to recommend help to others (12). They are also better able to choose timely and appropriate forms of treatment (13, 14). In contrast, adolescents with low levels of MHL show greater psychological distress (11, 15) and carry a higher risk for depression than those with adequate levels of MHL (16). There is demographic evidence that females tend to possess higher levels of MHL than males (17, 18) and that knowledge of somatic diseases and mental disorders among youth increases with age (19–21). However, since findings have been inconclusive regarding whether age and sex impact MHL [e.g., (22)], these variables should continuously be considered in MHL research.

While research evidence suggests varying MHL levels among different demographics, it is important to note that MHL levels among children and adolescents are generally considered to be low (16, 23). This is also true for COPMI, who often lack information and knowledge about mental illness in general (24, 25). They receive few factual explanations, have limited understanding of their parent’s mental disorder, and experience this as a difficult challenge (26, 27). COPMI need information about mental disorders (24, 26, 28, 29) and have expressed this need not only regarding knowledge about mental illness in general, but also regarding their parent’s specific disorder (28, 30, 31). Specific disorder knowledge is considered pivotal in aiding COPMI to adapt to the situation at hand and to improve their coping skills (24, 25). Parental behaviors related to the disorder can be considered illness related; thus, children can adjust their own behavior accordingly, manage negative feelings better, and less likely blame themselves for their parent’s disorder (24, 32). Additionally, greater knowledge is associated with more willingness to seek help for mental health problems (33). Providing COPMI with knowledge is, therefore, a means of equipping them with greater control and understanding of the mental disorder (34). Such increased understanding is thought to mediate the COPMI risk within the transgenerational transmission through sense making and the improved use of coping strategies (35, 36).

Working groups investigating knowledge in MHL have employed diverse methodologies, such as vignettes (8, 37), inductive qualitative content analysis (27, 31), questionnaires, and mixed methods (18, 36). However, there is a gap in the literature regarding COPMI’s uniform assessment of knowledge about their parents’ specific mental disorder across various common disorder groups in a large sample of COPMI (24, 25). Specifically, there are few suitable measures to assess knowledge of children and adolescents (38), and still fewer are directed toward COPMI specifically (39). Given the recommendation to incorporate qualitative methods to understand the perspectives of affected COPMI (26, 40), employing interviews with open-ended questions, such as those utilized in the COMPARE—family study (7), proves suitable for MHL analysis. To effectively capture nuanced levels of disorder knowledge, identify knowledge gaps, and facilitate comparisons across samples and time, we adopted a blended approach of inductive and deductive qualitative content analysis, integrating the symptom-based framework of the Diagnostic and Statistical Manual of Mental Disorders [DSM-5 (41)].

The assessment of COPMI’s disorder knowledge is highly relevant given MHL’s important role in promoting mental health in youth and the potentially mediating effect of disorder knowledge in the transgenerational transmission of mental disorders. However, COPMI’s knowledge about mental disorders in general, and their knowledge about the parental mental disorder specifically have not yet been assessed congruently in a large sample of COPMI. We aimed to address this research gap by assessing specific disorder knowledge among four frequently occurring disorder groups and by identifying potential demographic differences with regard to the level of knowledge. Our research questions were as follows:

1. What do children of parents with a mental disorder know about mental disorders in general?
2. What specific knowledge do children of parents with a mental disorder have about their parents’ specific disorder?
3. Are there age differences with respect to general and specific disorder knowledge?
4. Are there sex differences with respect to general and specific disorder knowledge?

## 2 Materials and methods

### 2.1 Design and ethical approval

To answer our research questions, we employed a qualitative analysis to assess COPMI’s disorder knowledge, and a quantitative analysis to detect differences within their knowledge. Ethical approval was granted by the Ethics Committee in the Department of Psychology of Philipps University Marburg; all participating children and their legal guardians provided written informed consent prior to study participation.

### 2.2 Procedure

MHL interviews were conducted with COPMI as part of the COMPARE—family study (7, 42). COMPARE—family is a preventive intervention for COPMI with the aim of interrupting the transmission of mental disorders in children of a parent with mental disorders. COMPARE—family was conducted as a multi-centered randomized controlled trial with two treatment arms in which families were assessed at four main measurement points. The inclusion criteria participating families had to meet were as follows: (1) a parent seeks outpatient psychotherapeutic care, (2) the parent currently meets diagnostic criteria for a DSM-5 disorder (41), and (3) the parent is caring for at least one child between the ages of 1.5 and 16 years. The exclusion criteria were as follows: (1) the parent is already in psychotherapeutic treatment, (2) the parent needs acute inpatient treatment (e.g., acute risk of committing suicide or acute psychosis), (3) all children fulfill the criteria for a severe mental illness and need prompt treatment, (4) the parent uses benzodiazepines continuously (intermittent drug use less than once every 2 weeks is allowed), or (5) the family has insufficient German language skills. Data used in this study were collected from the first assessment point (T1), which took place prior to the COMPARE—family treatment. At T1, a sociodemographic questionnaire was administered to assess household characteristics, and a structured clinical interview (DIPS) was conducted with the ill parent to assess mental disorders according to the DSM-5 (41), comorbid disorders, and disorder severity. Mental disorders in COPMI were assessed by administering a structured clinical interview (Kinder-DIPS) with one of the parents. Furthermore, at T1, the MHL interview was conducted face-to-face or via telephone with each participating family member. The interviews were conducted from 2018 to 2022 in the various COMPARE centers and lasted approximately 20 min. For analysis, the interviews were recorded and transcribed. If there were any ambiguities in the transcription, the transcription was checked and supplemented by a second person. Furthermore, randomly selected 5% of the transcribed interviews were cross-checked against the first version. We only analyzed the child version of the MHL interview here.

### 2.3 Participants

Data of N = 181 participants aged 5 to 17 years as a subsample of the COMPARE—family study were examined in the present study; detailed characteristics are presented in **Table 1**. Participants were classified as “child” when aged 5 to 11 years (n = 130) representing elementary school age. Participants were classified as “adolescent” when aged 12 to 17 years (n = 51) representing middle school and high school age. The children were on average *M* = 8.22 years old (*SD* = 1.79), and 66 were female (50.77%). The majority of the interviewed children were already going to school (n = 116, 89.23%), and 21 were diagnosed with a clinically relevant mental disorder at pre-assessment. No data were available from one child regarding the diagnosis of a mental disorder. Most children were interviewed about their parent’s Depressive Disorders or Anxiety Disorders. Two children were excluded from our analysis of specific disorder knowledge as they were interviewed about Depressive Disorders, while their parent’s primary diagnosis fell into the Anxiety Disorders or Somatic Symptom and Related Disorders. The adolescents were on average *M* = 13.43 years old (*SD* = 1.27), and 25 were female (49.02%). All were going to school; 11 were diagnosed with a clinically relevant mental disorder. Most adolescents were interviewed about their parent’s Depressive Disorders or Somatic Symptom and Related Disorders.

**Table 1:** Overview of participant characteristics.

	Child sample (n = 130)		Adolescent sample (n = 51)	
	M	SD	M	SD
Age (in years)	8.22	1.79	13.43	1.27
	n	%	n	%
Female sex	66	50.77	25	49.02
School attendance:	112	86.15	51	100.00
*Primary school*	88	67.69	0	0.00
*Secondary school*	24	18.46	51	100.00
Own clinical diagnosis^1^	23^a^	17.69^a^	11	21.57
Interviewed about:				
*Depressive Disorders*	49	37.69	23	50.00
*Anxiety Disorders*	42	32.30	7	15.22
*Trauma- and Stressor-Related Disorders*	23	17.69	7	15.22
*Somatic Symptom and Related Disorders*	6	4.61	9	19.56
Other disorders	10	7.69	5	9.80

^1^According to a structured interview (Kinder-DIPS) with a parent. ^a^N = 129.

### 2.4 Parents of the participants

Characteristics of the ill parent of the participating children and adolescents were taken into account. The characteristics of the other parent were not included in the analyses. Thus, a total of N = 148 parents with a mental illness were included in our sample; detailed characteristics are presented in **Table 2**. Twenty-one of the parents participated with two children and six participated with three children. The parents were on average *M* = 41.13 years old (*SD* = 6.88) with 110 of them being female (74.32%). Most were employed (83.56 %) and in a relationship (78.77 %); no data were obtained on employment and relationship status regarding two parents. Socioeconomic status was calculated as the family’s Highest International Socio-Economic Index [HISEI; (43)]. The HISEI indicates a family’s highest international socio-economic index (ISEI) value, which generally corresponds to the higher ISEI score of both parents. The HISEI can range from 16 to 90, and a high value corresponds to a high occupational status. The mean HISEI in the sample was *M* = 53.93 (*SD* = 17.95); HISEI data from five families were not available. The family’s mean HISEI of the present study slightly exceeds the average HISEI in the German PISA 2018 sample [=51.8, (44)] for comparison. The German PISA 2018 sample included more than 5,000 students, most of whom were 15 years old. Most of the parents’ primary diagnoses fell within the spectrum of Depressive Disorders, followed by Anxiety Disorders. The other diagnoses were distributed among Trauma- and Stressor-Related Disorders, Somatic Symptom and Related Disorders, and other disorders. Regarding disorder severity, we ignored subclinical values (0–3), while values between four and eight covered a moderate-to-severe spectrum. The diagnosis of most parents was estimated at level six or seven in severity; one value was not available. More than half showed clinically comorbid disorders; no data were available from two parents.

**Table 2:** Overview of characteristics of participant’s parents with mental illness.

	Parent sample	
	M	SD
Age (in years)	41.13	6.88
HISEI	53.93^a^	17.95^a^
	n	%
Female sex	110	74.32
Employment^1^	122^b^	83.56^b^
In a relationship^2^	115^b^	78.77^b^
Primary diagnosis^3^:		
*Depressive Disorders*	60	40.54
*Anxiety Disorders*	38	25.68
*Trauma- and Stressor-Related Disorders*	27	18.24
*Somatic Symptom and Related Disorders*	11	7.43
Other primary diagnosis	12	8.11
Severity of mental illness^3^:		
*4*	14^c^	9.52^c^
*5*	27^c^	18.37^c^
*6*	58^c^	39.46^c^
*7*	41^c^	27.89^c^
*8*	7^c^	4.76^c^
Clinical comorbid disorders^3^	84^b^	57.53^b^

^a^N = 143; ^b^N = 146; ^c^N = 147. ^1^part- and full-time work. ^2^Comprises being in a partnership or married, as opposed to being single, separated, divorced, or widowed. ^3^Assessed with the DIPS.

### 2.5 Measures

We conducted a semi-structured MHL interview to assess disorder knowledge. It is adapted from an interview, which was conducted as part of the psychoeducation intervention “Hope, Meaning, and Continuity” for families in which at least one parent has a depressive disorder (45). The MHL interview contains a total of 12 questions about mental disorders in general, knowledge about the ill parent’s primary diagnosis, causes of the parental mental disorder, dealing with it, and communication about the disorder. For this study’s analyses, we relied on only the first six questions displayed in **Table 3**. First, all children were asked about what they knew about mental illness in general, and then disorder-specific knowledge was collected from the children in line with the parent’s primary diagnosis. Children were also asked whether their parents had a mental disorder or other problems with their feelings currently or in the past. However, not every child was asked every interview question due to the correct and incorrect application of skipping rules by the different study center’s interviewer. To specify, the application of correct skipping rules provided for skipping from question 2 to 6, if a child indicated that he or she had no knowledge about the primary diagnosis. If question 6 was negated as well, the interviewer could skip to question 11. Incorrect application of skipping rules led, for example, to the interviewer skipping from question 2 directly to question 11, leaving out question 6.

**Table 3:** Overview of interview questions used for the analyses.

No.	Question
1	What do you think is a mental illness?
2	What is an X*?
3	If someone had an X*, how would he or she behave?
4	How/By which aspects would you notice that someone has an X*?
5	What are the symptoms of an X*?
6	Has either of your parents ever had an X* or other difficulties with his/her feelings?

X*, parental primary diagnosis inserted here.

### 2.6 Qualitative analyses

We analyzed the qualitative content of the transcribed MHL interviews following qualitative research guidelines (46) using the software MAXQDA (47). First, LK and MS determined the aim of the category construction on the basis of the research questions regarding which general and specific disorder knowledge COPMI have. Furthermore, the type of categories and level of abstraction were determined with a mixed form of content structuring and evaluative qualitative analysis with a high level of abstraction. In the next step, LK screened half of the interviews to become familiar with the material. The size of the segments to be coded was determined as partial sentences, sentences, or short paragraphs, and then the sequential analysis was implemented via a per-interview approach. Initially, LK defined seven higher-order categories relying on the interview structure. A randomly selected sample of 35% of the interviews was then coded *in vivo* by LK until the category system was saturated. During the subsequent systematization and organization of these inductively developed categories, we discovered a strong similarity with the DSM-5 disorder criteria (41). Following consultation with MS and HC, the inductively developed categories were substituted by deductive categories. These comprise the disorder-specific definitions and diagnostic criteria as described in the DSM-5 (41) and are matched to the initial inductive categories. In addition to these DSM-5-oriented deductive categories, three additional categories (no knowledge, correct conceptions, incorrect conceptions) were added to prevent loss of information. The developed category system was then applied by LK to 50% of the material to test its applicability to the data set. LK then developed a coding book and analyzed the whole set of interviews. To verify the quality of the coding system and determine interrater reliability, KP additionally analyzed 25% of the interviews independently using the coding book. KP was blinded for the codes that were assigned to the material by LK. Interrater reliability was estimated using Cohen’s kappa (κ) within the corresponding function in MAXQDA.

### 2.7 Quantitative analyses

Chi-square tests of independence were applied to identify associations between COPMI’s age and sex with general and specific disorder knowledge. Associations were tested at a significance level α of 5%. Odds ratios (OR) were calculated as effect sizes. To better align with the assumption of chi-square tests, which stipulates that expected frequencies should exceed 5 in at least 80% of the cells (48), we combined the two primary categories of “no knowledge” and “incorrect conceptions” given the limited number of participants displaying exclusively incorrect conceptualizations. Three levels of knowledge groups resulted in our analyzing in terms of age and sex: “no knowledge,” “good conceptualization,” and “good disorder knowledge.” The group “no knowledge” included all COPMI who displayed either no knowledge or only incorrect conceptions. The group “good conceptualization” included all COPMI who displayed either only correct conceptions or a mixture of correct conceptions and incorrect ones. The group “good disorder knowledge” included all COPMI displaying disorder knowledge that was also in line with DSM-5 descriptions (41) regardless of any additional display of incorrect or correct conceptualizations. This was implemented for both general and specific disorder knowledge. For general disorder knowledge, Pearson’s chi-square values are reported, as the assumptions of chi-square tests were met. For specific disorder knowledge, maximum likelihood ratio chi-square values are reported, as the described assumption of chi-square tests was not met.

## 3 Results

In this section, the categorical system with its structure of higher-order, main, and subcategories is described first. The first and second higher-order categories are then displayed separately: general disorder knowledge and whether the child knew about the parental disorder. Next is an overview of the higher-order categories 3 to 6, the specific disorder knowledge. Following this, the specific disorder’s main and subcategories are reported. Then, the last higher-order category and quantitative results are presented. We report results for children first and then for adolescents, and illustrate results with quotes of the participants.

### 3.1 Categorical system

An overview of the categorical system is displayed in **Table 4**. The system consists of three category levels: higher-order categories, **main categories** and *subcategories*. The developed higher-order categories include the COPMI’s knowledge about mental disorders in general (category 1), whether the interviewee knew that his or her parent was ill (category 2), specific knowledge about the parent’s disorder (categories 3–6), and specific knowledge about additionally required disorder criteria (category 7). Four main categories were developed for the higher-order categories of general and specific disorder knowledge (categories 1 and 3–6): **no knowledge** (x**.1**), **incorrect conceptions** (x**.2**), **correct conceptions** (x**.3**), and **general/specific disorder knowledge** (x.**4**).

**Table 4:** Overview of the developed categorical system.

*No.*	Higher-order categories, main categories, and subcategories	Subcategory descriptions; *Interview segment was coded if the child described:*
*1*	Knowledge about mental disorders in general	
	**1.1 No knowledge**	
	**1.2 Incorrect conceptions**	
	**1.3 Correct conceptions**	
	**1.4 General disorder knowledge**	
	*1.4.1 Syndrome*	*That mental disorders are defined by several mental symptoms occurring simultaneously or lists several symptoms*
	*1.4.2 Dysfunction/illness*	*Mental illness as a (significant) problem, disease or disorder, or as something that is dysfunctional or malfunctioning*
	*1.4.3 Affected psychological functioning*	*Mental illness in connection with thoughts, behavior, or feelings*
	*1.4.4 Psychobiological functioning*	*Mental disorders against the background of psychological, biological, or development-related processes or sees them as being rooted in these processes. This includes an association with psychological or mental functions and the localization in the mind*
	*1.4.5 Distress/impairment*	*Mental disorders associated with significant suffering or significant impairment*
	*1.4.6 Deviation from the norm*	*Mental disorders as something that is not normatively expected or culturally recognized as a reaction to a common stressor. Alternatively, the child describes that mental disorders are not defined by socially deviant behaviors, such as those of a cultural or political nature, and do not refer to individual social conflicts*
*2*	Knowledge about whether the parent is ill	
	**2.1 Illness not known to the child**	
	**2.2 Known diagnosis or illness suspected**	
	**2.3 Not clear**	
*3*	Knowledge about Depressive Disorders	
	**3.1 No knowledge**	
	**3.2 Incorrect conceptions**	
	**3.3 Correct conceptions**	
	**3.4 Specific disorder knowledge**	
	*3.4.1 Psychosomatic alterations*	*That a depressive disorder can be identified by somatic, affective, or cognitive changes in the affected person*
	* 3.4.2 Irritable mood*	*Those affected as angry, irritable, aggressive, or very annoyed*
	* 3.4.3 Depressed mood*	*Symptoms of depression (e.g., a negative view of themselves and the world) or that those affected are sad, cry, or feel empty, desperate, or hopeless*
	* 3.4.4 Anhedonia*	*Depressive disorders in terms of reduced interest or reduced pleasure in things or activities on part of the affected person*
	* 3.4.5 Weariness*	*Depressive disorders with tiredness, exhaustion, and lack of energy in the affected person or describes observations of psychomotor slowness or restlessness*
	* 3.4.6 Sleep/appetite change*	*Associations of depressive disorders with symptoms of sleep disturbances (insomnia, hypersomnia) or a change in appetite or weight in the affected person*
	* 3.4.7 Low self-esteem*	*A connection to reduced self-confidence, feelings of guilt, or other feelings of worthlessness or dissatisfaction regarding the person with the disorder*
	* 3.4.8 Impaired executive functioning*	*Depressive disorders with difficulties or problems in thinking, concentration, and decision making*
	* 3.4.9 Thoughts about death*	*That depressed individuals have (recurring) thoughts of death*
*4*	Knowledge about Anxiety Disorders	
	**4.1 No knowledge**	
	**4.2 Incorrect conceptions**	
	**4.3 Correct conceptions**	
	**4.4 Specific disorder knowledge**	
	* 4.4.1 Anxiety/panic response*	*Various forms of panic, anxiety, or fear of something (place, person, situation) or a reaction to this feeling*
	* 4.4.2 Avoidant–cautious behavior*	*Avoiding or fleeing from situations or behaving in a generally cautious, fearful or avoidant manner as a specific behavior in the context of anxiety disorders*
	* 4.4.3 Behavior abnormalities*	*That the affected person shows conspicuous, strange, or maladjusted behavior*
	* 4.4.4 Somatic symptoms*	*Physiological or somatic symptoms of the affected person in the context of anxiety disorders*
	* 4.4.5 Negative thoughts*	*Affected individuals are very worried, have negative thoughts or fearful expectations*
	* 4.4.6 Dissociation or nightmares*	*That the affected person has nightmares or describes symptoms that resemble derealization or depersonalization of said person*
*5*	Knowledge about Trauma- and Stressor-Related Disorders	
	**5.1 No knowledge**	
	**5.2 Incorrect conceptions**	
	**5.3 Correct conceptions**	
	**5.4 Specific disorder knowledge**	
	* 5.4.1 Critical event*	*The presence of a traumatic or stressful factor, event, or situation*
	* 5.4.2 Avoidance*	*That the patient avoids certain things, places, people, and situations, sometimes persistently or for a long time*
	* 5.4.3 Diminished involvement*	*That the affected person shows reduced interest or participation in important activities*
	* 5.4.4 Dissociation or intrusion*	*That affected individuals have symptoms of re-experiencing traumatic or stressful situations, have repeated intrusions, or dissociate*
	* 5.4.5 Negative cognitions*	*That the affected person has negative thoughts*
	* 5.4.6 Negative affect*	*That the disorder is accompanied by anhedonia, negative affect, or a negative mood*
	* 5.4.7 Increased arousal*	*That the affected person shows increased alertness or attention or has an increased level of arousal*
*6*	Knowledge about Somatic Symptom and Related Disorders	
	**6.1 No knowledge**	
	**6.2 Incorrect conceptions**	
	**6.3 Correct conceptions**	
	**6.4 Specific disorder knowledge**	
	* 6.4.1 Stressful somatic symptom*	*That the affected individual has a stressful somatic or physiological illness or corresponding symptoms or pain*
	* 6.4.2 Excessive thoughts*	*That those affected think extensively, very strongly, or excessively about the distressing symptoms or the illness*
	* 6.4.3 Negative feelings*	*Negative feelings of the affected person in connection with the physiological or somatic illness or symptoms*
	* 6.4.4 Symptom-related behavior*	*That those affected exhibit specific behaviors that are related to the presenting symptoms*
	* 6.4.5 Adverse psycho-behavioral impact*	*That the affected person exhibits certain behavior that adversely affects the state of health or describes corresponding psychological factors that negatively influence the person’s health status*
*7*	Knowledge about additionally required disorder criteria	
	**7.1 Time criterion**	
	**7.2 Criterion of distress or impairment**	
	**7.3 Exclusion criterion of differential diagnoses**	

Higher-order categories are highlighted in gray, main categories are shown in bold, and subcategories are shown in italics.

The main categories **no knowledge** (x**.1**), **incorrect conceptions** (x**.2**), and **correct conceptions** (x**.3**) have no further subcategories. COPMI who did not answer or stated that they lacked knowledge were assigned to the **no knowledge** category. Inappropriate or incorrect mentions were assigned to the category of **incorrect conceptions**. Appropriate, correct, and exemplary descriptions exceeding any DSM-5-descriptions were assigned to the category of **correct conceptions**. For **general/specific disorder knowledge**, *subcategories* were developed. For **general disorder knowledge** (**1.4**), these subcategories (*1.4.1* to *1.4.6*) are in line with the DSM’s definition of a mental disorder (41). For **specific disorder knowledge** (**3.4**; **4.4**; **5.4**; **6.4**), subcategories (*3.4.1–3.4.9*; *4.4.1–4.4.6*; *5.4.1–5.4.7*; *6.4.1–6.4.5*) are in line with the specific DSM-5 disorder definitions and main diagnostic criteria (41). Three additionally required disorder criteria (category 7) were assessed together across all the specific disorders surveyed, namely, the time criterion, the criterion of distress or impairment, and the exclusion criterion of differential diagnoses. These represent the diagnostic criteria listed at the end of each disorder in the DSM-5 (41). The interrater reliability across all ratings was moderate according to McHugh (49) with κ = 0.67.

### 3.2 Knowledge about mental disorders in general

**Figure 1:**
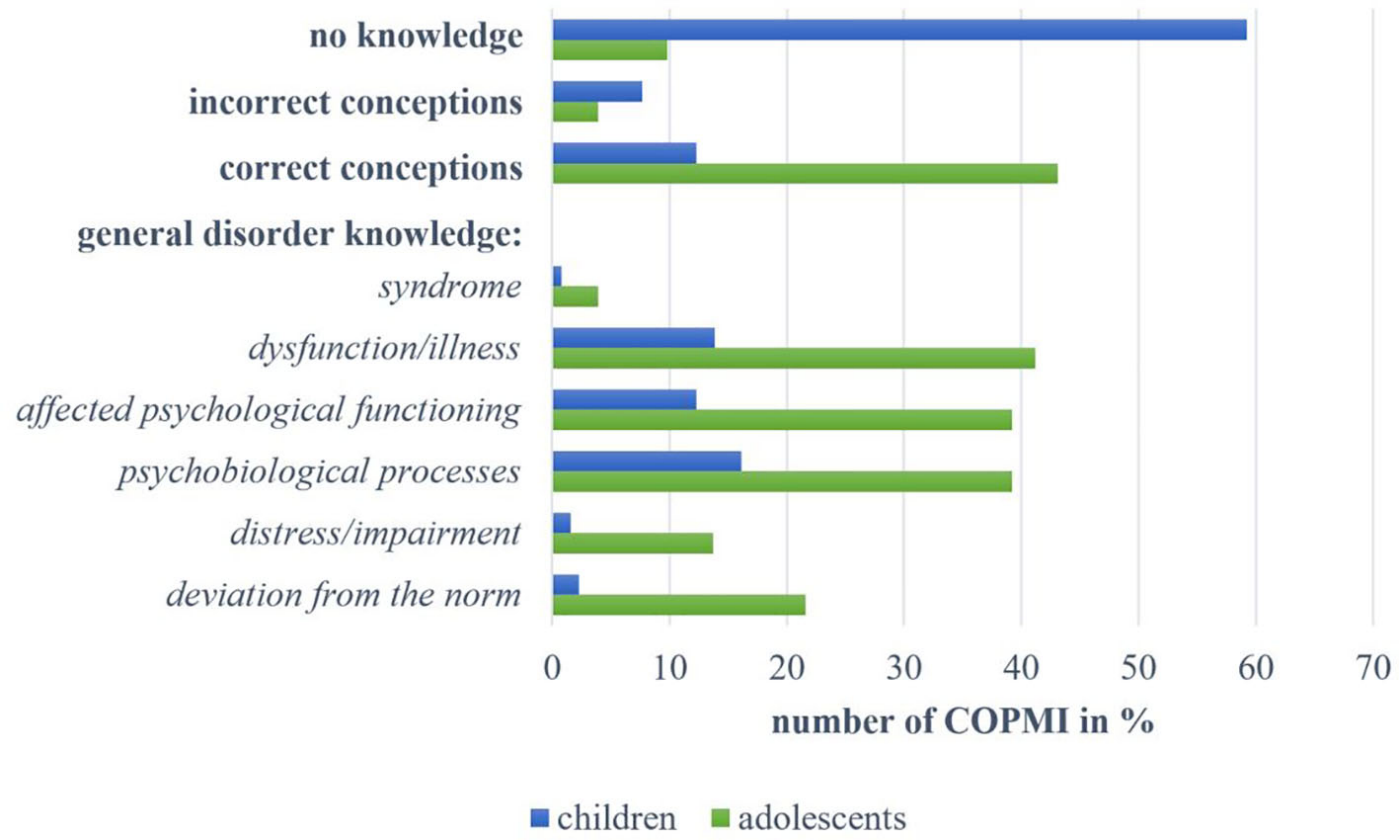
Overview of answers by children and adolescents within the categories on knowledge about mental disorders in general.

The majority of interviewed children (N = 77, 59.23%) showed **no knowledge** of mental illness in general, as can be seen in **Figure 1**. Mentions from 10 children (7.69%) were assigned to the category of **incorrect conceptions**, for instance, describing mental disorders as something “where one has no real illness, but rather that it’s the hormones” (10-year-old female, C9) or “if you can’t speak German properly” (11-year-old male, C10). Incorrect conceptions also included misconceptions about mentally ill people (“that’s those in wheelchairs or so”; 8-year-old male, C11). Sixteen children (12.31%) revealed **correct conceptions** of the construct of mental disorders providing example diagnoses. Regarding **general disorder knowledge**, the following three aspects were mentioned most frequently: 21 children (16.15%) associated mental illness with *psychobiological processes*, for example, as “something in the brain, like a faulty circuit in the brain” (11-year-old female, C1) or as “some change in the genes” (9-year-old male, C2). Eighteen (13.85%) could describe it as *dysfunction or illness*, for example, as “something like a disease” (8-year-old male, C3) or “that you have some problems and can't handle them like the other people around you” (11-year-old female, C4). Sixteen children (12.31%) described *affected psychological functioning* with most of them referring to affected emotions like “when you are very sad” (9-year-old male, C5) or “that someone is angry” (8-year-old male, C6). Beyond that, mentions from three children (2.31%) were assigned to the category “*deviation from the norm*” and two described associated *distress or impairment* (“if you can't do it like someone who is perhaps completely healthy”; 11-year-old female, C7). We assigned one child’s description of mental disorders as a *syndrome* (“Depression, for example, can be many things: grief, anger, anxiety, nausea, dizziness”; 8-year-old female, C8).

Among the adolescents, five (9.80%) displayed **no knowledge**. The mentions of two adolescents (3.92%) were assigned to the category of **incorrect conceptions**. They described that mental disorders were “not really an illness” (14-year-old male, A5). Almost half of all adolescents (N = 22, 43.14%) revealed **correct conceptions**. These comprised example diagnoses and adequate descriptions (“that you have an altered impression on your environment”; 13-year-old female, A9). Meanwhile, regarding **general disorder knowledge**, most adolescents associated mental disorders with one of the following three aspects: 21 (41.18%) described it as *dysfunction or illness*. Twenty adolescents (39.22%) associated mental disorders with *psychobiological processes*, like “a change of character [ … ] in the brain” (14-year-old male, A1), “if you have problems that are psychological in nature” (13-year-old female, A2) or “where the head tends to go a bit crazy from time to time” (12-year-old male, A3). The same number of adolescents (39.22%) described *affected psychological functioning* as follows: emotions were most frequently described as “when you don’t feel so well” (12-year-old female, A4), and behavior was referred to as being unusual, “peculiar” (14-year-old male, A1) or altered. Mentions of 11 adolescents (21.57%) were assigned to the category “*deviation from the norm*”, for example “if a person’s behavior deviates from the ‘norm’” (14-year-old male, A5) or “this is when you perhaps behave differently than, let's say, normal. There is no such thing as normal, but there is such a thing as not behaving in a way that would be good for other people either” (15-year-old female, A6). Associated *distress or impairment* was mentioned by seven adolescents (13.73%) in the form of “problems [ … ] that can affect your health” (13-year-old female, A7) or “so that you can no longer live properly” (14-year-old male A8). Two adolescents (3.92%) described mental disorders as a *syndrome*.

### 3.3 Knowledge about whether the parent is ill

Of all COPMI, n = 118 children were asked whether their parent was ill. Almost 67% did **not know that their parent had a mental disorder** (n = 79, 66.95%), while one quarter of the children **suspected** some form of disorder **or knew about it** (n = 30, 25.42%), like this 7-year-old boy (C12): “Yes, I have a feeling about it. I just think there’s something not right with my parents”. For n = 9 children, it **was unclear** whether they knew, suspected, or did not know about the parental mental disorder due to the child’s answer’s unclear wording or due to biased wording of the question (“And do you think, for example, that your parents were ever really sad?”; interviewer I1). The remaining n = 63 children in the sample were not asked the corresponding question.

In sum, n = 50 adolescents were interviewed on whether their parent was ill. While n = 23 (46%) of them **did not know about their parent’s disorder**, n = 27 (54%) **suspected or knew about** some form of mental disorder. One adolescent was not asked the corresponding question.

### 3.4 Knowledge about the specific parental mental disorder

#### 3.4.1 Summarized specific disorder knowledge

**Figure 2:**
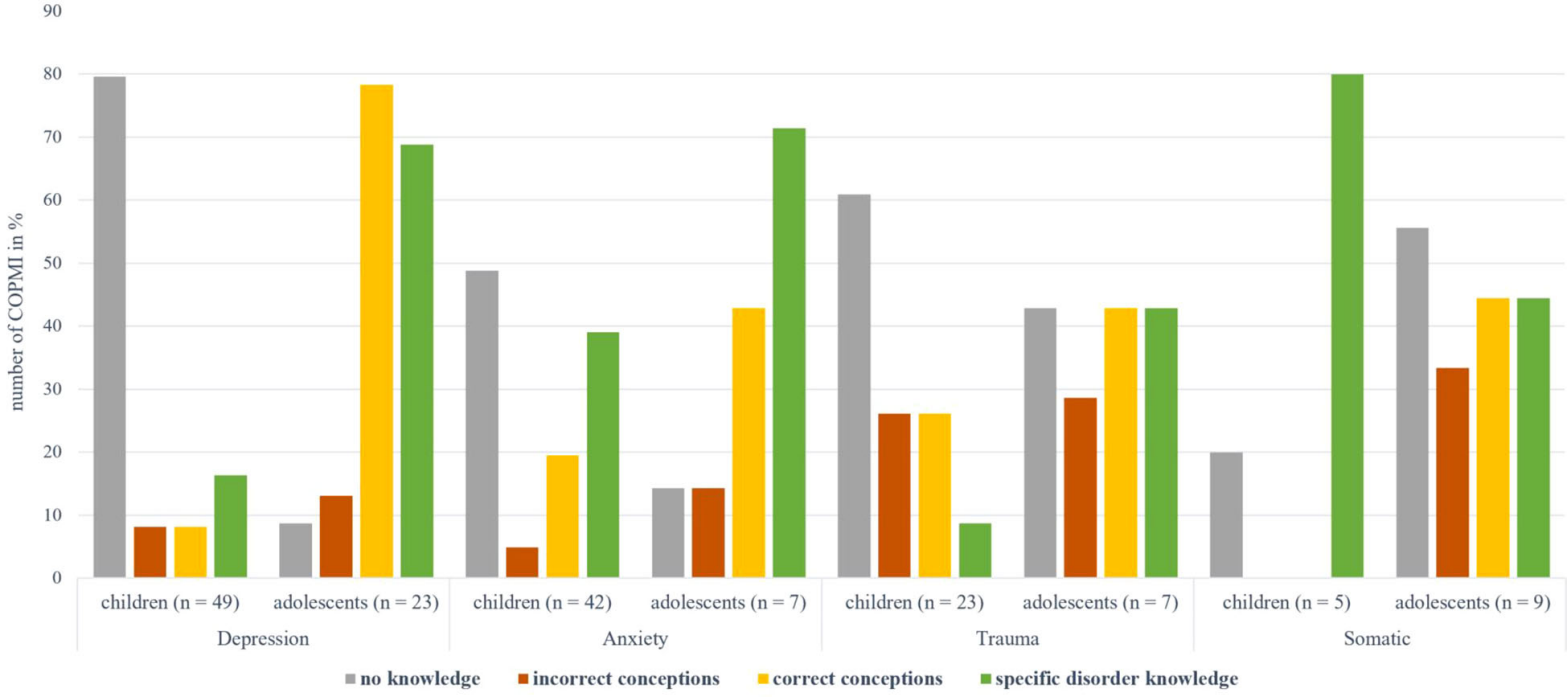
Overview of the main categories of specific knowledge by children and adolescents for the different disorder groups. Depression: Depressive Disorders, anxiety: Anxiety Disorders, trauma: Trauma- and Stressor-Related Disorders, somatic: Somatic Symptom and Related Disorders.

For a better overview, **Figure 2** illustrates the four main categories of specific knowledge (no knowledge, incorrect conceptions, correct conceptions, and specific disorder knowledge) assessed within the different specific disorder groups separately for children and adolescents. This is followed by a split overview of the results from each of the four disorder groups.

#### 3.4.2 Knowledge about Depressive Disorders

**Figure 3:**
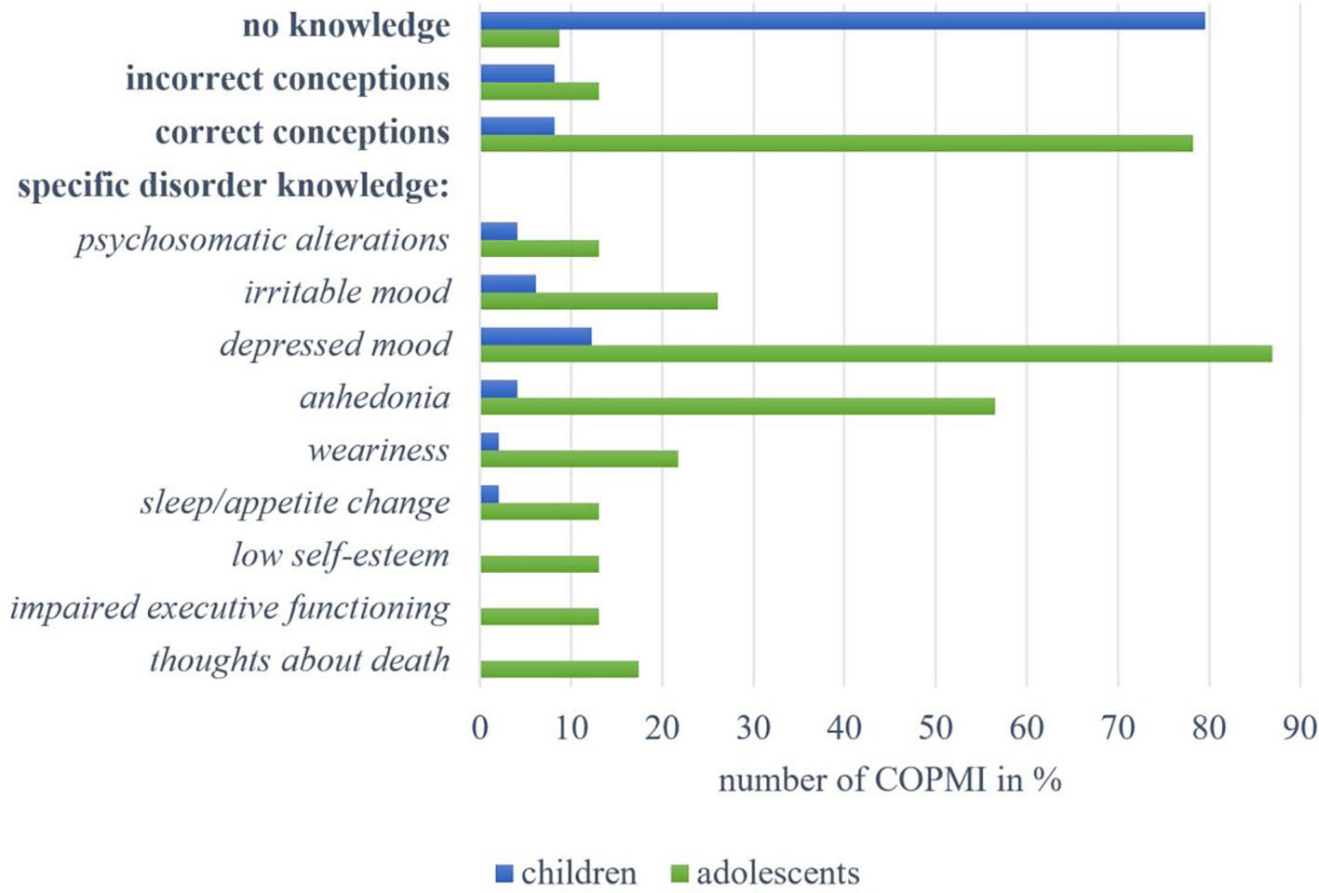
Overview of answers by children and adolescents within the categories on knowledge about Depressive Disorders.

A total of n = 49 children were interviewed about their knowledge of Depressive Disorders. As seen in **Figure 3**, almost 80% (n = 39, 79.59%) reported **no knowledge**. Four children displayed **incorrect conceptions**, such as confusing symptoms of depressive disorders with those of an infectious disease (“uh, like Corona”, 8-year-old male, C2). Likewise, four children displayed **correct conceptions**, such as “he [ … ] seldom talks” (11-year-old female, C14). Regarding **specific disorder knowledge**, six children (12.24%) described *depressed mood* in the form of “that you feel helpless, and desperate” (11-year-old female, C1) or “that's when people are somehow really sad because of a certain thing. And not just once, but for a long time and over and over again and very strongly” (11-year-old female, C13). Three children (6.12%) mentioned *irritable mood*, and two children’s descriptions (4.08%) could be assigned to the category of *anhedonia* (“He just doesn't enjoy many things as much as he used to,” 11-year-old female, C1). Two children (4.08%) described *psychosomatic alterations*, and one child (2.04%) described *weariness*. No mentions could be assigned to the following categories: *thoughts about death*, *impaired executive functioning*, and *low self-esteem*.

Furthermore, n = 23 adolescents were interviewed on their knowledge about Depressive Disorders. Only two of them (8.70%) displayed **no knowledge**. Three adolescents (13.04%) had **incorrect conceptions** of the disorder, for instance, naming “laziness” (12-year-old male, A14) as a disorder symptom. Up to 80% of the adolescents (n = 18, 78.26%) displayed **correct conceptions** of depressive disorders. These include descriptions of co-occurring somatic symptoms and observed behavior, for example, “that there is something that is bothering the person, but somehow you also realize that they don't want to or can't talk about it like that” (17-year-old female, A16). At the same time, one adolescent adequately stated: “I feel like you often can’t really see it. I’ve noticed that people often just put on a fake smile” (16-year-old female, A10). Regarding **specific disorder knowledge**, most frequently mentioned (n = 20, 86.96%) was *depressed mood*, like “feeling empty, being unhappy with life” (16-year-old female, A10), “always seeing something bad in everything” (14-year-old male, A11) or “when you give up on yourself” (15-year-old male, A12). More than half of the adolescents mentioned aspects of *anhedonia* (n = 13, 56.52%), describing it as “being unmotivated” (16-year-old female, A13) or when one “doesn't feel like doing almost anything” (12-year-old male, A14). Six adolescents (26.09%) reported *irritable mood*, and five (21.74%) described *weariness*. Four adolescents (17.39%) mentioned *thoughts about death*. Other mentioned symptoms, each named by three adolescents (26.09%), comprise *sleep or appetite changes*, *low self-esteem*, and *impaired executive functioning*. Three adolescents also described *psychosomatic alterations* (“for example, when you’ve gotten to know someone who’s cheerfully and suddenly you realize that they rarely laugh anymore”; 13-year-old male, A15).

#### 3.4.3 Knowledge about Anxiety Disorders

**Figure 4:**
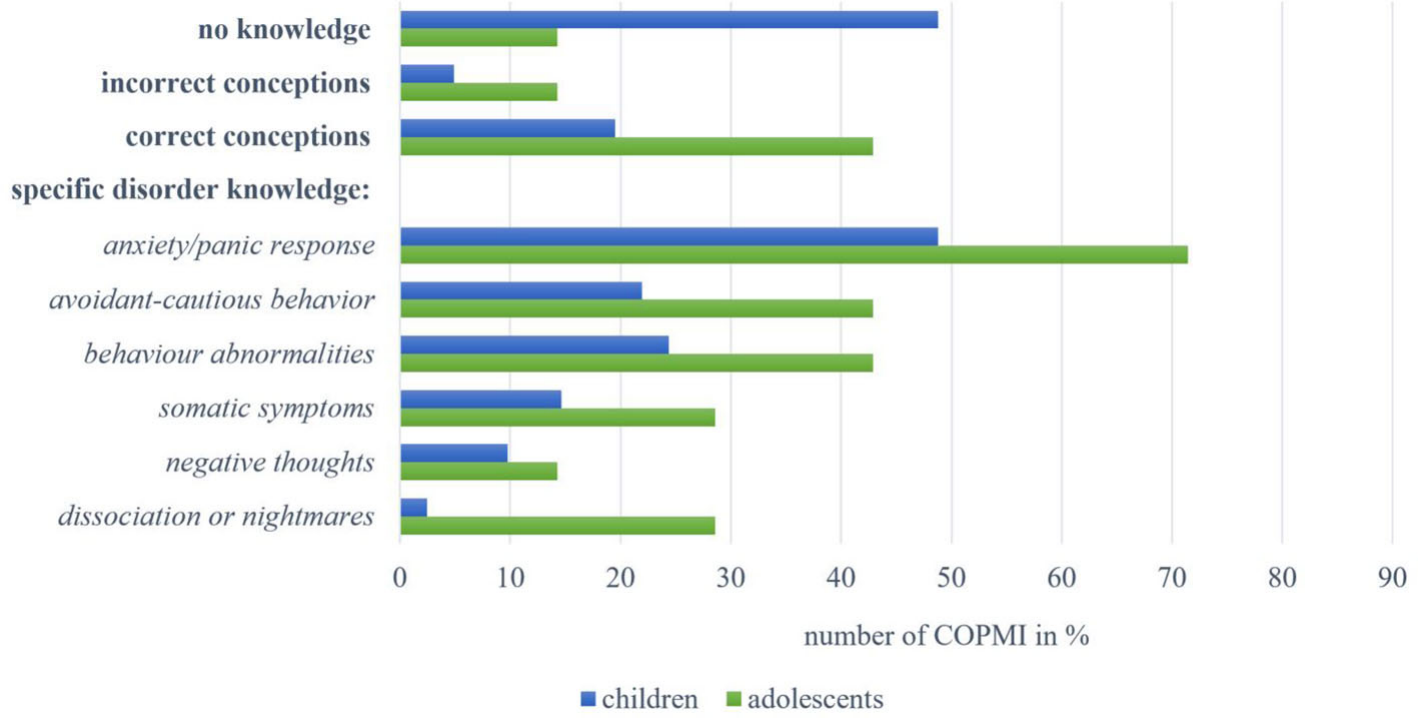
Overview of answers by children and adolescents within the categories on knowledge about Anxiety Disorders.

A total of n = 41 children were interviewed on their knowledge about Anxiety Disorders. As seen in **Figure 4**, almost half of them (n = 20, 48.78%) reported **no knowledge** of the disorder. Two children (4.88%) held **incorrect conceptions**, for example, naming “extortion” as a disorder symptom (9-year-old male, C18). Eight children (19.51%) revealed **correct conceptions** that mainly referred to facial expressions as disorder symptoms or descriptions of specific situations. Regarding **specific disorder knowledge**, almost half of the children (n = 20, 48.78%) described an *anxiety or panic response*, such as “if you panic right away when you're somewhere [ … ] and that’s like a disorder, too” (8-year-old female, C15). Ten children (24.39%) reported on *behavioral abnormalities*. Nine children (21.95%) described *avoidant-cautious behavior*, such as “he gets so anxious and [ … ] then he just wants to get out” (9-year-old female, C16) or “that he pulls away from everything” (9-year-old male, C17). Six children (14.63%) mentioned *somatic symptoms* like trembling or sweating. *Negative thoughts* were mentioned by four children, and one child described symptoms of *dissociation or nightmares*.

In addition, seven adolescents were interviewed on their knowledge about Anxiety Disorders. One (14.29%) reported **no knowledge,** and one adolescent’s **incorrect conception** referred to being “stupid” (15-year-old male, A20). **Correct conceptions** of three adolescents (42.86%) included “having claustrophobia” (12-year-old female, A18) and “perhaps also [being] a little sad” (14-year-old female, A19). Meanwhile, regarding **specific disorder knowledge**, five adolescents (71.43%) named an *anxiety or panic response*. Knowledge on *avoidant–cautious behavior* (n = 3, 42.86%) included mentions of being “very hesitant” (13-year-old female, A2). Descriptions of *behavioral abnormalities* (n = 3, 42.86%) referred to strange or restless behavior. Further reports on symptoms included *somatic symptoms* (n = 2, 28.57%) as well as symptoms of *dissociation or nightmares* (n = 2, 28.57%; “if one’s no longer really responsive, I’d say”; 14-year-old male, A8). One adolescent (14.29%) mentioned *negative thoughts*.

#### 3.4.4 Knowledge about Trauma- and Stressor-Related Disorders

**Figure 5:**
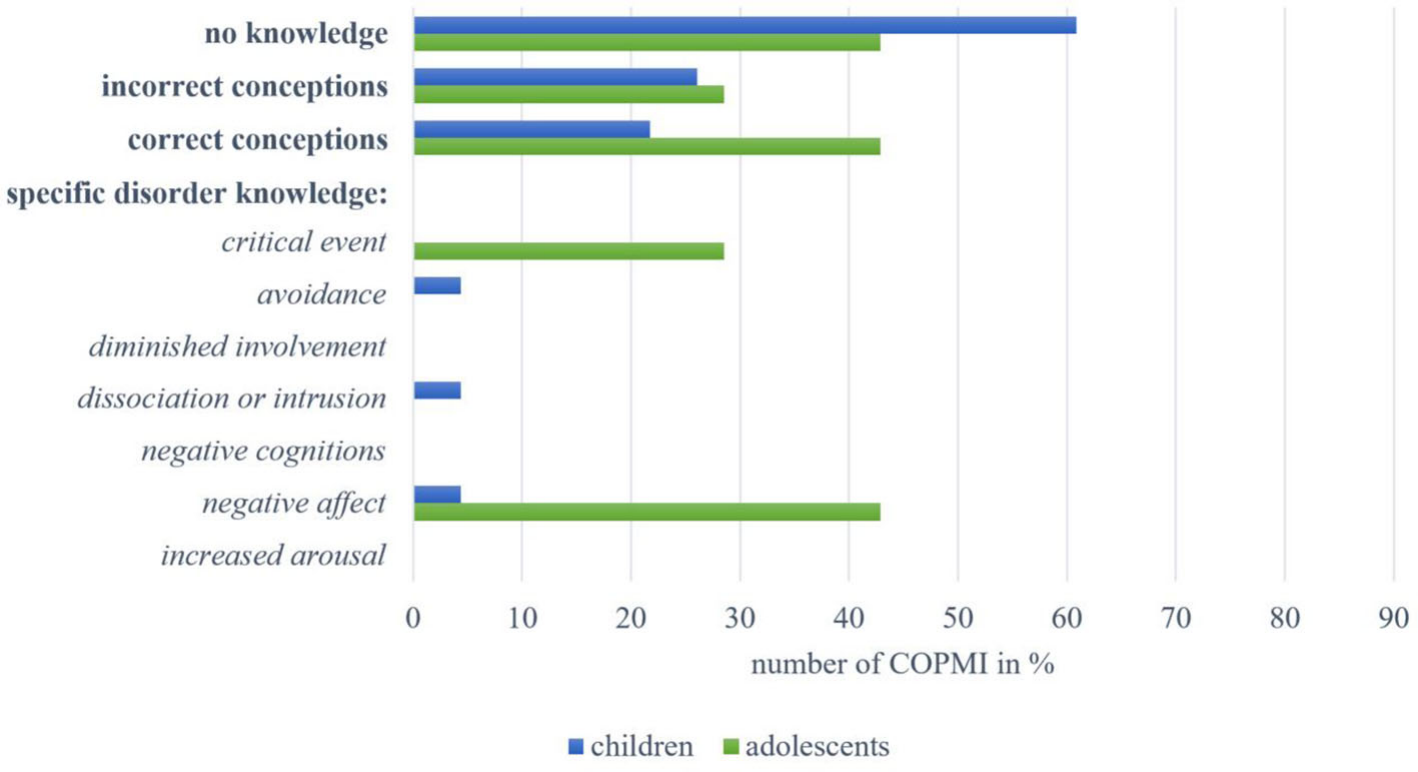
Overview of answers by children and adolescents within the categories on knowledge about Trauma- and Stressor-Related Disorders.

Among all children, n = 23 were interviewed on their knowledge about Trauma- and Stressor-Related Disorders. Fourteen children (60.87%) showed **no knowledge** of the disorder, as seen in **Figure 5**. Six children (26.09%) displayed **incorrect conceptions**, referring among other things to being “an outsider” (8-year-old male, C24), wanting to “fit in with everyone” (8-year-old male, C25) or “when you bother a king” (5-year-old female, C26). Five children (21.74%), however, held **correct conceptions**, including “that one reacts strangely” (10-year-old female, C22) or “maybe it's that you can't really return to being normal” (8-year-old male, C23). Regarding **specific disorder knowledge**, we assigned one child’s statement (4.35%) to *dissociation or intrusion* (“so you can see it [ … ] sometimes that they look really scared”; 8-year-old female, C30), one to *negative affect* (“when you’re sad”; 10-year-old female, C20), and one to *avoidance* (“some of them often run away”; 8-year-old female, C21). None of the interviewed children’s mentions could be assigned to the categories: *critical event*, *diminished involvement*, *negative cognitions*, or *increased arousal*.

Among all adolescents, seven were interviewed on their knowledge about Trauma- and Stressor-Related Disorders. Three (42.86%) reported **no knowledge**. Two adolescents (28.57%) held **incorrect conceptions**, one of whom referred to having “problems fitting into a group [and being] uncooperative” (15-year-old female, A22). Three adolescents (42.86%) displayed **correct conceptions**, “that you get insomnia or something” (13-year-old male, A21), when “you’re lonely” (15-year-old female, A22), or when “it’s all too much” (14-year-old male, A23). Meanwhile, regarding **specific disorder knowledge**, three adolescents (42.86%) described *negative affect* and two (28.57%) named the existence of a *critical event*. No statements from the interviewed adolescents could be assigned to the categories: *avoidance*, *diminished involvement*, *dissociation or intrusion*, or *negative cognitions*.

#### 3.4.5 Knowledge about Somatic Symptom and Related Disorders

**Figure 6:**
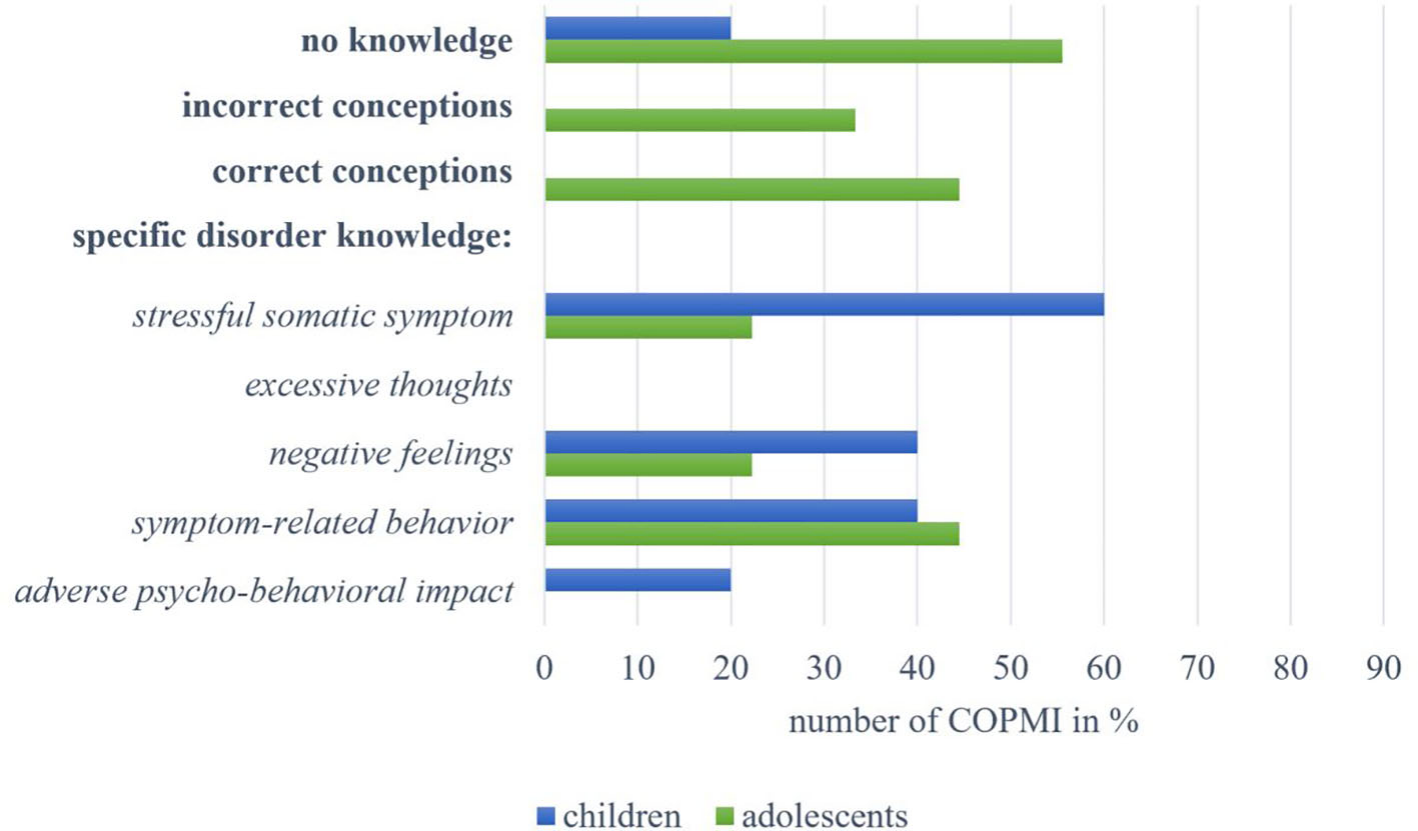
Overview of answers by children and adolescents within the categories on knowledge about Somatic Symptom and Related Disorders.

Five children were interviewed on their knowledge about Somatic Symptom and Related Disorders. One (20.00%) reported **no knowledge**, as seen in **Figure 6**, while no mentions of these children could be assigned to the categories of **incorrect** or **correct conceptions**. Regarding **specific disorder knowledge**, three children (60.00%) named the existence of a *stressful somatic symptom*. Two children each (40.00%) named *negative feelings* (“when he feels sad”; 6-year-old female, C27) and *symptom-related behaviors* (“then mom lies on the sofa or in bed”; 6-year-old female, C28). We allocated one child’s description (20.00%) to the category of *adverse psycho-behavioral impact*: “And then we tell her to lie down. But she doesn't want to lie down because she’s always got something to do. So she can't just sit there and watch the others while they’re doing something. That's no option for her” (11-year-old female, C4). None of the interviewed children’ mentions could be assigned to the *excessive thoughts* category.

We also interviewed nine adolescents on their knowledge about Somatic Symptom and Related Disorders. Five (55.56%) reported **no knowledge**, and three adolescents (33.33%) held **incorrect conceptions**, such as describing the symptoms as “pain [ … ] that doesn’t appear when it should” (12-year-old male, A26). Four of the interviewed adolescents (44.44%) revealed **correct conceptions**, for example, being “more easily irritable” (14-year-old female, A24). Likewise, regarding **specific disorder knowledge**, four adolescents (44.44%) described *symptom-related behaviors*, such as “when you try to take it easy on yourself” (14-year-old female, A24) or “when they withdraw a bit” (13-year-old female, A25). Two adolescents each (22.22%) described the existence of a *somatic symptom* and mentioned *negative feelings* (“[being] very often sad”; 13-year-old female, A25). We were unable to assign any statements to the categories of *excessive thoughts* or *adverse psycho-behavioral impact*.

#### 3.4.6 Specific knowledge about additionally required disorder criteria

We included all interviews with adolescents (n = 51) and n = 128 interviews with children to assess the specific knowledge about additionally required disorder criteria. The **time criterion** was assigned most frequently, as three children and 12 adolescents described a certain time intensity or duration for the specific disorders. Their statements mostly concerned descriptions within the spectrum of depressive disorders (n = 14) like this explanation by an 11-year-old girl (C1): “when [ … ] there’s no getting around the fact that you’re just sad all the time.” The **criterion of distress or impairment,** for instance as in “[one] can no longer live their everyday life properly” (12-year-old female, A27), was observed in interviews with three children and five with adolescents. We were unable to assign any mentions to the **excluded differential diagnoses criterion**.

## 4 Quantitative analyses

### 4.1 Age and sex differences regarding general and specific disorder knowledge

Separate chi-square tests for independence revealed significant associations between COPMI’s age and sex with general and specific disorder knowledge. First, age was significantly associated with general disorder knowledge, χ^2^ (2) = 41.84, p < 0.001. The odds of displaying general disorder knowledge versus no knowledge were 15.26 times higher among adolescents than children. Similarly, the odds of revealing good conceptions rather than no knowledge were 19.68 times higher among adolescents than children. Second, age proved to be significantly associated with specific disorder knowledge, Lχ^2^ (2) = 22.90, p < 0.001. The odds of showing specific disorder knowledge rather than none were 5.74 times higher among adolescents than children. Similarly, the odds of exhibiting specific disorder knowledge rather than a good conceptualization were 7.77 times higher in adolescents than children. Third, COPMI’s sex was significantly associated with general disorder knowledge, χ^2^ (2) = 7.05, p < 0.05. The odds of showing general disorder knowledge versus no knowledge were 1.94 times higher in males than in females. Similarly, the odds of displaying good conceptions rather than no knowledge were 3.96 times higher in males than in females. COPMI’s sex was not significantly associated with specific disorder knowledge, Lχ^2^ (2) = 0.44, p = 0.819.

## 5 Discussion

MHL plays an essential role in promoting mental health in children and adolescents, exerting significant influence on their help-seeking behavior and thus serving as a pivotal factor in mitigating the perpetuation of psychopathological symptoms in youth. Within the MHL concept, disorder knowledge is of particular importance among youth populations carrying an increased risk for developing a mental disorder, such as COPMI, as it is considered a mediator in the transgenerational transmission of mental disorders. To further determine the role of disorder knowledge, the aim of the current study was to identify COPMI’s disorder knowledge in general and their knowledge about the parental disorder specifically. We also aimed to identify differences in disorder knowledge in terms of COPMI’s age and sex.

With regard to research question 1 on COPMI’s knowledge about mental disorders in general, we found that most children did not report general disorder knowledge. This finding is in line with research showing a lack of knowledge and missing factual information among COPMI (27). Nevertheless, several of our study’s children displayed some disorder knowledge, and correct conceptualizations were observed more often than incorrect ones. This evidence illustrates that children are certainly capable of adequately appraising and describing what mental disorders constitute—in contrast to parents often feeling that their children are too young to talk about the issue (16). Adolescents meanwhile demonstrated both knowledge in line with the DSM-5 and correct conceptions beyond the DSM-5 diagnostic definitions. Previous studies indicated low MHL levels among youth (16, 23), as well as inadequate or inaccurate knowledge among COPMI (24, 25), and might have underestimated the knowledge levels of young people for methodological reasons. These include the common use of specific case vignettes (16, 23) or the qualitative analysis of focus group interviews or online forum content, whereas we asked the children directly and in a one-to-one setting.

With regard to research question 2 on COPMI’s specific knowledge about their parents’ specific mental disorder, we demonstrated that the level of knowledge was dependent on the disorder group about which the COPMI were being interviewed. Children interviewed on parental Depressive Disorders or Trauma- and Stressor-Related Disorders most frequently revealed no knowledge. For Anxiety Disorders, half of the interviewed children lacked knowledge, and within the Somatic Stress Disorders, children showed noticeably high levels of specific disorder knowledge in line with the DSM-5. It is important to note that children may not have consciously acquired knowledge about depression, anxiety disorders, or trauma disorders (16, 26). However, they may be able to associate somatic symptoms or the parents’ visible pain more easily with somatic disorders. Moreover, correct conceptions were infrequently exhibited by children across any disorder group, suggesting that children may either possess specific learned knowledge about a disorder, or lack knowledge altogether. Maladaptive parental behavior resulting from the disorder could be misattributed by the child due to a lack of knowledge resulting in a negative development where the child associates the parent’s behavior with its own behavior rather than with the disorder [e.g. (6, 29)]. Adolescents interviewed about a parental Depressive Disorder or Anxiety Disorder frequently displayed specific disorder knowledge in line with and beyond the DSM-5. As COPMI often are uninformed about the parental disorder (31), adolescents might have consciously encountered aspects of depression or anxiety disorders additionally through interactions with peers or social media, thus acquiring correct symptom knowledge and a correct conceptualization of the disorders. Similarly, Miles et al. (50) identified individuals with high MHL as having more experience with disorders through peer history than do individuals with less MHL. The high prevalence of mood disorders during adolescence supports this notion (51). Among the adolescents interviewed on Trauma- and Stressor-Related Disorders, almost half displayed no knowledge, and a third held incorrect conceptions. Symptom knowledge only encompassed two aspects of the disorder, but it was supplemented by a correct conceptualization of the disorder. For Somatic Symptom and Related Disorders, adolescents most frequently showed no knowledge. This finding stands in contrast to the children’s results but may be attributable to the fact that children were asked more often about a “pain disorder,” whereas adolescents were asked more often about a “somatic stress disorder”—thus the use of different terms already introduced a bias.

Taken together, we found that children often demonstrated no specific disorder knowledge, except for somatic disorders, and that adolescents showed moderate-to-high levels of symptom knowledge about their parent’s specific disorder. This finding suggests that children, in particular, need both more information on the subject of mental disorders, in general, and additional information on their parent’s specific illness. Furthermore, our study data align with the subjective need expressed by COPMI for more information about their parents’ specific disorder (27) highlighting that a significant portion of COPMI lack specific disorder knowledge altogether. To rule out the possibility that interviewed children are unable to understand the interview questions due to their developmental level, it would be beneficial to consider strategies for more adequately recording the knowledge of younger children.

Regarding research question 3, we have demonstrated significant associations between COPMI’s age and their general and specific disorder knowledge. Just as previous research has revealed (21), so too did our results indicate a significant association between COPMI’s age and the level of knowledge. The odds of possessing both correct general and specific disorder knowledge were significantly higher in adolescents than in children. This increase of knowledge with age during youth is already well established (20, 21) and was confirmed regarding general and specific knowledge in our explorative study. There is also no research account to date showing that parents communicate with children under the age of 11 years about their illness (52). Our data confirms this lack of communication, namely, that most children were unaware of their parent’s mental disorder. Together, these findings contribute to explaining the limited knowledge COPMI have about mental disorders despite being (unknowingly) confronted with the impact of mental disorders in the family setting on a day-to-day basis.

With regard to research question 4, we were able to show a significant association between COPMI’s sex and general disorder knowledge. The odds of having correct general disorder knowledge were significantly higher in males than in females. In contrast to findings cited by Campos et al. (18) and many others, our study reveals more correct disorder knowledge in males than in females in both age groups. We assume that this finding might be due to the novel approach we took. As females are considered to be more sensitive to mental health issues (18), their recognition of illness symptoms in case of vignettes may trigger different conclusions than we drew in the COMPARE—family study. As we found that males were more likely to display general knowledge, a lesson from our findings may be that interventions should support males in applying their knowledge, such as recognizing symptoms in others, and to foster knowledge in females for the protective effects of MHL. As sex was not significantly associated with specific disorder knowledge, the role of sex in MHL expression should be examined further. In this context, it would also be beneficial to examine the sex dyads between child and ill parent in more detail given that more mentally ill mothers than fathers were included in our sample. This could potentially influence sex-specific attribution patterns as well as the communication about mental illness within the family.

Taken together, the fact that we took a deductive approach enabled us to assess COPMI’s knowledge about mental disorders in general and about the parental mental disorder specifically. Moreover, the use of a system with the four main categories (no knowledge, symptom knowledge in line with the DSM, correct conceptions, and incorrect conceptions), allowed for a nuanced assessment of disorder knowledge. This forms an important foundation for subsequently establishing a relationship between the level of disorder knowledge and parameters in the transgenerational transmission model. In particular, the association between disorder knowledge and the development of psychopathologies in COPMI might be determined this way.

### 5.1 Strengths and limitations

One of the major strengths of our study is our sample’s heterogeneity supporting the transferability of our findings. This heterogeneity is characterized by the multi-centered approach, the broad age range of included COPMI, consideration of different parental diagnoses, comorbidities and degrees of disorder severity, as well as different sex dyads between COPMI and affected parent. Nonetheless, the extent of transferability of our sample’s findings to COPMI in general may be limited. As the COMPARE—family study required parents to be willing to undergo psychotherapy and regularly attend study appointments, our findings could be biased by an overrepresented group of COPMI with highly motivated parents. Different findings might have yielded for COPMI outside the care system. However, as our sample’s COPMI displayed a lack of knowledge of mental disorders, we can assume that COPMI outside the care system would have even lower levels of knowledge. It is also worth noting that all types of mental disorders were considered in the COMPARE—family study, but not all COPMI could be accounted for in our analysis of specific disorder knowledge, as the disorders were considered on a group level. The conclusiveness of our results can therefore not be generalized to all types of mental disorders, and the disorder groups underrepresented in this study should be investigated further. Nevertheless, the large sample size is one of the strengths of our study, which enabled a comprehensive comparison of disorder knowledge.

Another study limitation is the moderate interrater reliability of the code system we developed. To use the deductive approach more broadly, a higher level of agreement between coders should be aimed at. This can be achieved by revising the codebook and a second coder’s training. Meanwhile, a major strength of this study lies in our deductive approach, which enabled us to capture the presence and extent of disorder knowledge. Previous studies on knowledge in MHL have utilized vignettes (8, 37), inductive qualitative content analysis (27, 31), questionnaires, and mixed methods (18, 36). However, some of these methods have limitations. For instance, symptom recognition in vignettes does not equate to knowledge and is viewed critically (53, 54). Furthermore, MHL questionnaires are still being developed (18) and do not include the specific parental diagnosis. Moreover, assessing correct knowledge and comparing results within the inductive approach remains problematic because of different methodologies (24). To address some of the aforementioned methodological limitations, we took a novel qualitative approach in this study. By applying the DSM-oriented deductive qualitative content analysis, knowledge levels could be determined congruently in line with different forms of knowledge (general and specific) and among different samples (children and adolescents; various disorder groups). Findings generated in this way have the potential to indicate which variables should be targeted in MHL interventions aiming to strengthen COPMI’s disorder knowledge.

### 5.2 Implications for research and practice

Practical implications of the present study include the consideration of our findings in MHL interventions for COPMI. For example, we demonstrated that males displayed more general knowledge about disorders and that they might lack the skills to apply that knowledge. We also showed that the two age groups had different levels of specific knowledge regarding the different specific disorders. Drawing from our findings, we recognize a great need for psychoeducation among younger children whose parents suffer from depressive disorders. The lack of knowledge was most prevalent in this subgroup, while at the same time, depression is one of the most common mental disorders affecting a large number of families. Since targeting knowledge as an intervention may have limited effects on actual behavioral change (55), it might be beneficial to consider integrating psychoeducational aspects of MHL with mental health action components as, for example, proposed by Marinucci et al. (56).

Research implications from the present work arise from pending research on the relationship between COPMI’s disorder knowledge and coping strategies as well as the development of child psychopathology in the transmission model. Other research implications include the analysis of associations between the child’s, their ill parent’s, and their healthy parent’s disorder knowledge. We, therefore, suggest that the DSM-oriented deductive qualitative content analysis approach be retained, optimized, and applied to other samples and additional measurement time points within the COMPARE—family study and beyond.

## 6 Conclusion

Knowledge about mental disorders is viewed as an important coping mechanism for COPMI. In particular, it helps in dealing with the family situation on a day-to-day basis and thus may increase the sensitivity to the development of COPMI’s own symptoms. Disorder knowledge, therefore, has the potential to play an important role in preventing the development of COPMI’s psychopathologies, if it is targeted right. In our study, we showed that COPMI have limited knowledge about mental disorders in general and about the specific parental disorder. This evidence, however, proved to be dependent on COPMI’s age, partly dependent on their sex, and on the parental disorder group. Strengthening COPMI by enhancing their knowledge and understanding of the specific parental disorder in an action-focused way should be a major goal of future preventive efforts. Moreover, an important focus of MHL research in COPMI should be to shed light on the role of disorder knowledge in the transgenerational transmission of mental disorders. The methodological approach we propose can, therefore, be considered as a template to determine the knowledge component of MHL in COPMI in a comparable manner across different populations, disorder groups, and over time.

## Data availability statement

The datasets presented in this article are not readily available because of patient confidentiality and legal restrictions. Requests to access the datasets should be directed to HC, hanna.christiansen@staff.uni-marburg.de.

## Ethics statement

The studies involving humans were approved by Ethics Committee in the Department of Psychology of Philipps University Marburg. The studies were conducted in accordance with the local legislation and institutional requirements. Written informed consent for participation in this study was provided by the participants’ legal guardians/next of kin.

## Author contributions

LK: Conceptualization, Formal analysis, Methodology, Writing – original draft. KP: Formal analysis, Writing – review & editing. MS: Conceptualization, Methodology, Writing – review & editing. CS: Funding acquisition, Project administration, Writing – review & editing. MK: Funding acquisition, Project administration, Writing – review & editing. KO: Funding acquisition, Project administration, Writing – review & editing. CR: Funding acquisition, Project administration, Writing – review & editing. RS: Funding acquisition, Project administration, Writing – review & editing. LW: Funding acquisition, Project administration, Writing – review & editing. A-LZ: Funding acquisition, Project administration, Writing – review & editing. HC: Funding acquisition, Methodology, Project administration, Supervision, Writing – review & editing.

## References

[1] Solmi M Radua J Olivola M Croce E Soardo L Salazar de Pablo G. Age at onset of mental disorders worldwide: large-scale meta-analysis of 192 epidemiological studies. Mol Psychiatry. (2022) 27:281–95. doi: 10.1038/s41380-021-01161-7 PMC8960395 34079068

[2] Erskine HE Moffitt TE Copeland WE Costello EJ Ferrari AJ Patton G. A heavy burden on young minds: the global burden of mental and substance use disorders in children and youth. Psychol Med. (2015) 45:1551–63. doi: 10.1017/S0033291714002888 PMC5922255 25534496

[3] Kessler RC Foster CL Saunders WB Stang PE. Social consequences of psychiatric disorders, I: Educational attainment. Am J Psychiatry. (1995) 152:1026–32. doi: 10.1176/ajp.152.7.1026 7793438

[4] van Santvoort F Hosman CMH Janssens JMAM van Doesum KTM Reupert A van Loon LMA. The impact of various parental mental disorders on children's diagnoses: A systematic review. Clin Child Fam Psychol Rev. (2015) 18:281–99. doi: 10.1007/s10567-015-0191-9 26445808

[5] Uher R Pavlova B Radua J Provenzani U Najafi S Fortea L. Transdiagnostic risk of mental disorders in offspring of affected parents: a meta-analysis of family high-risk and registry studies. World Psychiatry. (2023) 22:433–48. doi: 10.1002/wps.21147 PMC10503921 37713573

[6] Hosman CMH van Doesum KTM van Santvoort F. Prevention of emotional problems and psychiatric risks in children of parents with a mental illness in the Netherlands: I. The scientific basis to a comprehensive approach. Aust e-Journal Advancement Ment Health. (2009) 8:250–63. doi: 10.5172/jamh.8.3.250

[7] Christiansen H Reck C Zietlow A-L Otto K Steinmayr R Wirthwein L. Children of mentally III parents at risk evaluation (COMPARE): design and methods of a randomized controlled multicenter study-part I. Front Psychiatry. (2019) 10:128. doi: 10.3389/fpsyt.2019.00128 30971958 PMC6443700

[8] Jorm AF Korten AE Jacomb PA Christensen H Rodgers B Pollitt P. “Mental health literacy”: a survey of the public's ability to recognise mental disorders and their beliefs about the effectiveness of treatment. Med J Aust. (1997) 166:182–6. doi: 10.5694/j.1326-5377.1997.tb140071.x 9066546

[9] Kutcher S Wei Y Costa S Gusmão R Skokauskas N Sourander A. Enhancing mental health literacy in young people. Eur Child Adolesc Psychiatry. (2016) 25:567–9. doi: 10.1007/s00787-016-0867-9 27236662

[10] Zhang J-Y Ji X-Z Zhou Y-Q. The mediating effect of mental health literacy on psychological resilience and psychological distress of medical college students. Perspect Psychiatr Care. (2023) 2023:1–7. doi: 10.1155/2023/3461121

[11] Zhang X Yue H Hao X Liu X Bao H. Exploring the relationship between mental health literacy and psychological distress in adolescents: A moderated mediation model. Prev Med Rep. (2023) 33:102199. doi: 10.1016/j.pmedr.2023.102199 37223554 PMC10201844

[12] Coles ME Coleman SL. Barriers to treatment seeking for anxiety disorders: initial data on the role of mental health literacy. Depress Anxiety. (2010) 27:63–71. doi: 10.1002/da.v27:1 19960488

[13] Wright A Jorm AF Harris MG McGorry PD. What's in a name? Is accurate recognition and labelling of mental disorders by young people associated with better help-seeking and treatment preferences? Soc Psychiatry Psychiatr Epidemiol. (2007) 42:244–50. doi: 10.1007/s00127-006-0156-x 17450404

[14] Freţian AM Graf P Kirchhoff S Glinphratum G Bollweg TM Sauzet O. The long-term effectiveness of interventions addressing mental health literacy and stigma of mental illness in children and adolescents: systematic review and meta-analysis. Int J Public Health. (2021) 66:1604072. doi: 10.3389/ijph.2021.1604072 34975363 PMC8714636

[15] Pehlivan Ş Tokur Kesgi NM Uymaz P. Psychological distress and mental health literacy in university students. Perspect Psychiatr Care. (2021) 57:1433–41. doi: 10.1111/ppc.12709 33330978

[16] Lam LT. Mental health literacy and mental health status in adolescents: a population-based survey. Child Adolesc Psychiatry Ment Health. (2014) 8:26. doi: 10.1186/1753-2000-8-26

[17] Cotton SM Wright A Harris MG Jorm AF McGorry PD. Influence of gender on mental health literacy in young Australians. Aust N Z J Psychiatry. (2006) 40:790–6. doi: 10.1080/j.1440-1614.2006.01885.x 16911755

[18] Campos L Dias P Palha F Duarte A Veiga E. Development and psychometric properties of a new questionnaire for assessing mental health literacy in young people. Univ Psychol. (2016) 15:61. doi: 10.11144/Javeriana.upsy15-2.dppq

[19] Sigelman C Maddock A Epstein J Carpenter W. Age differences in understandings of disease causality: AIDS, colds, and cancer. Child Dev. (1993) 64:272–84. doi: 10.1111/j.1467-8624.1993.tb02909.x 8436034

[20] Myant KA Williams JM. Children's concepts of health and illness: understanding of contagious illnesses, non-contagious illnesses and injuries. J Health Psychol. (2005) 10:805–19. doi: 10.1177/1359105305057315 16176958

[21] Fox C Buchanan-Barrow E Barrett M. Children's understanding of mental illness: an exploratory study. Child Care Health Dev. (2008) 34:10–8. doi: 10.1111/j.1365-2214.2007.00783.x 18171438

[22] Lee HY Hwang J Ball JG Lee J Albright DL. Is health literacy associated with mental health literacy? Findings from Mental Health Literacy Scale. Perspect Psychiatr Care. (2020) 56:393–400. doi: 10.1111/ppc.12447 31736081

[23] Attygalle UR Perera H Jayamanne BDW. Mental health literacy in adolescents: ability to recognise problems, helpful interventions and outcomes. Child Adolesc Psychiatry Ment Health. (2017) 11:38. doi: 10.1186/s13034-017-0176-1 28814972 PMC5557470

[24] Cudjoe E Chiu MY. What do children know about their parent’s mental illness? A systematic review of international literature on children in families with mental illness. Children Youth Serv Rev. (2020) 119:105638. doi: 10.1016/j.childyouth.2020.105638

[25] Gladstone BM Boydell KM Seeman MV McKeever PD. Children's experiences of parental mental illness: a literature review. Early Interv Psychiatry. (2011) 5:271–89. doi: 10.1111/eip.2011.5.issue-4 21883973

[26] Mordoch E. How children understand parental mental illness: “you don't get life insurance. What's life insurance?”. J Can Acad Child Adolesc Psychiatry. (2010) 19:19–25. 20119563 PMC2809442

[27] Trondsen MV. Living with a mentally ill parent: exploring adolescents' experiences and perspectives. Qual Health Res. (2012) 22:174–88. doi: 10.1177/1049732311420736 21873281

[28] Grove C Reupert A Maybery D. Gaining knowledge about parental mental illness: how does it empower children? Child Family Soc Work. (2013) 20:377–86. doi: 10.1111/cfs.12086

[29] Cogan N Riddell S Mayes G. The understanding and experiences of children affected by parental mental health problems: a qualitative study. Qual Res Psychol. (2005) 2:47–66. doi: 10.1191/1478088705qp024oa

[30] Zeighami R Oskouie F Joolaee S. Mental health needs of the children of parents with mental illness. J Nurs Midwifery Sci. (2018) 5:95. doi: 10.4103/JNMS.JNMS_36_18

[31] Valiakalayil A Paulson L Tibbo P. Burden in adolescent children of parents with schizophrenia. Soc Psychiatry Psychiatr Epidemiol. (2004) 39:532. doi: 10.1007/s00127-004-0778-9 15243690

[32] Dam K Hall EOC. Navigating in an unpredictable daily life: a metasynthesis on children's experiences living with a parent with severe mental illness. Scand J Caring Sci. (2016) 30:442–57. doi: 10.1111/scs.12285 26763757

[33] Rickwood DJ Deane FP Wilson CJ. When and how do young people seek professional help for mental health problems? Med J Aust. (2007) 187:S35–9. doi: 10.5694/j.1326-5377.2007.tb01334.x 17908023

[34] Reupert AE Maybery D. ”Knowledge is power“: educating children about their parent's mental illness. Soc Work Health Care. (2010) 49:630–46. doi: 10.1080/00981380903364791 20711943

[35] Grove C Riebschleger J Bosch A Cavanaugh D van der Ende PC. Expert views of children's knowledge needs regarding parental mental illness. Children Youth Serv Rev. (2017) 79:249–55. doi: 10.1016/j.childyouth.2017.06.026

[36] Riebschleger J Costello S Cavanaugh DL Grové C. Mental health literacy of youth that have a family member with a mental illness: outcomes from a new program and scale. Front Psychiatry. (2019) 10:2. doi: 10.3389/fpsyt.2019.00002 30778305 PMC6369184

[37] Coles ME Ravid A Gibb B George-Denn D Bronstein LR McLeod S. Adolescent mental health literacy: young people's knowledge of depression and social anxiety disorder. J Adolesc Health. (2016) 58:57–62. doi: 10.1016/j.jadohealth.2015.09.017 26707229

[38] Wei Y Hayden JA Kutcher S Zygmunt A McGrath P. The effectiveness of school mental health literacy programs to address knowledge, attitudes and help seeking among youth. Early Interv Psychiatry. (2013) 7:109–21. doi: 10.1111/eip.12010 23343220

[39] Riebschleger J Grové C Cavanaugh D Costello S. Mental health literacy content for children of parents with a mental illness: thematic analysis of a literature review. Brain Sci. (2017) 7:4. doi: 10.3390/brainsci7110141 PMC5704148 29072587

[40] Gladstone BM Boydell KM McKeever P. Recasting research into children's experiences of parental mental illness: beyond risk and resilience. Soc Sci Med. (2006) 62:2540–50. doi: 10.1016/j.socscimed.2005.10.038 16316714

[41] American Psychiatric Association. (2013). Diagnostic and statistical manual of mental disorders. (5th ed.). doi: 10.1176/appi.books.9780890425596

[42] Stracke M Gilbert K Kieser M Klose C Krisam J Ebert DD. COMPARE family (Children of mentally ill parents at risk evaluation): A study protocol for a preventive intervention for children of mentally ill parents (Triple P, evidence-based program that enhances parentings skills, in addition to gold-standard CBT with the mentally ill parent) in a multicenter RCT-part II. Front Psychiatry. (2019) 10:54. doi: 10.3389/fpsyt.2019.00054 30873047 PMC6401604

[43] Ganzeboom HB de Graaf PM Treiman DJ. A standard international socio-economic index of occupational status. Soc Sci Res. (1992) 21:1–56. doi: 10.1016/0049-089X(92)90017-B

[44] Reiss K Weis M Klieme E Köller O. PISA 2018. Grundbildung im internationalen Vergleich. Waxmann (2019) Münster. doi: 10.31244/9783830991007

[45] Beardslee WR ed. Hoffnung, Sinn und Kontinuität: Ein Programm für Familien depressiv erkrankter Eltern. Tübingen: Dgvt-Verl (2009). p. 198.

[46] Kuckartz U Rädiker S. Qualitative inhaltsanalyse. Methoden, praxis, computerunterstützung: grundlagentexte methoden. Weinheim, Basel: Beltz Juventa (2022). p. 274.

[47] VERBI Software. MAXQDA, Software für qualitative Datenanalyse. Berlin, Deutschland: Consult. Sozialforschung GmbH (1989 – 2023).

[48] McHugh ML. The chi-square test of independence. Biochem Med (Zagreb). (2013) 23:143–9. doi: 10.11613/issn.1846-7482 PMC3900058 23894860

[49] McHugh ML. Interrater reliability: the kappa statistic. Biochem Med. (2012), 22(3):276–82. doi: 10.11613/issn.1846-7482 PMC3900052 23092060

[50] Miles R Rabin L Krishnan A Grandoit E Kloskowski K. Mental health literacy in a diverse sample of undergraduate students: demographic, psychological, and academic correlates. BMC Public Health. (2020) 20:1699. doi: 10.1186/s12889-020-09696-0 33187487 PMC7663887

[51] Silva SA Silva SU Ronca DB Gonçalves VSS Dutra ES Carvalho KMB. Common mental disorders prevalence in adolescents: A systematic review and meta-analyses. PloS One. (2020) 15:e0232007. doi: 10.1371/journal.pone.0232007 32324835 PMC7179924

[52] Mueller J Callanan MM Greenwood K. Communications to children about mental illness and their role in stigma development: an integrative review. J Ment Health. (2016) 25:62–70. doi: 10.3109/09638237.2015.1021899 26207330

[53] O’Dell L Crafter S de Abreu G Cline T. The problem of interpretation in vignette methodology in research with young people. Qual Res. (2012) 12:702–14. doi: 10.1177/1468794112439003

[54] Sai G Furnham A. Identifying depression and schizophrenia using vignettes: a methodological note. Psychiatry Res. (2013) 210:357–62. doi: 10.1016/j.psychres.2013.05.004 23712044

[55] Albarracín D Fayaz-Farkhad B Granados Samayoa JA. Determinants of behaviour and their efficacy as targets of behavioural change interventions. Nat Rev Psychol. (2024) 3:377–92. doi: 10.1038/s44159-024-00305-0 PMC12407158 40909436

[56] Marinucci A Grové C Allen K-A Riebschleger J. Evaluation of a youth mental health literacy and action program: Protocol for a cluster controlled trial. Ment Health Prev. (2021) 24:200216. doi: 10.1016/j.mhp.2021.200216

